# TWO-YEAR COURSE OF WALKING ADAPTABILITY IN PERSONS LIVING WITH LATE EFFECTS OF POLIO

**DOI:** 10.2340/jrm.v56.14727

**Published:** 2024-03-18

**Authors:** Jana TUIJTELAARS, Merel-Anne BREHM, Jos W. R. TWISK, Frans NOLLET

**Affiliations:** 1 Department of Rehabilitation Medicine, Amsterdam UMC location University of Amsterdam, Amsterdam; 2 Amsterdam Movement Sciences, Rehabilitation & Development, Amsterdam; 3 Amsterdam UMC location Vrije Universiteit Amsterdam, Department of Epidemiology and Data Science, Amsterdam, The Netherlands

**Keywords:** poliomyelitis, falls, walking adaptability, leg-muscle strength, follow-up

## Abstract

**Objective:**

To evaluate the 2-year course of walking adaptability in persons with late effects of polio.

**Design:**

Prospective cohort study.

**Patients:**

A total of 48 persons with late effects of polio (69% female, mean age 63.1 years) with a fall history and/or fear of falling.

**Methods:**

Walking adaptability (i.e. variable target-stepping and reactive obstacle-avoidance) was assessed on an interactive treadmill at baseline, 1 year and 2 years. Further, leg-muscle strength and balance were assessed at baseline. The course of walking adaptability was analysed with linear mixed models. Based on median values, subgroups were defined for low vs high baseline walking-adaptability and for clinical characteristics. Tme by subgroup interactions were analysed.

**Results:**

Variable target-stepping and reactive obstacle-avoidance did not change (*p* > 0.285). Reactive obstacle-avoidance improved for persons with a high balance score at baseline (*p* = 0.037), but not for those with lower scores (*p* = 0.531). No other time by subgroup interactions were found (*p* > 0.126).

**Conclusion:**

Walking adaptability did not change in persons with late effects of polio over 2 years, and walking adaptability course did not differ between subgroups stratified for walking adaptability determinants, except for balance. Since falls are a major problem among persons with late effects of polio, future studies should investigate whether walking adaptability declines over a longer time and which persons are most at risk.

LAY ABSTRACT

Falls are related to a reduced walking adaptability in persons with late effects of polio, which may deteriorate over time due to late onset polio sequelae. This study investigated whether walking adaptability in persons with late effects of polio deteriorates over time. To this end, 48 participants performed walking adaptability tests on an interactive treadmill at baseline, 1 year and 2 years. Walking adaptability outcomes were assessed and baseline outcomes were compared with outcomes at year 2. Reactive obstacle avoidance improved for persons with a high balance score. Furthermore, walking adaptability did not change in 2 years, possibly due to the absence of a decline in clinical characteristics known to be associated with walking adaptability (e.g. leg-muscle strength). Given the slow decline in leg-muscle strength as one of the best predictors for walking adaptability, future studies should investigate whether walking adaptability declines over a longer time period.

After several decades of stable functioning that follows an acute polio infection, many persons with late effects of polio are confronted with new or increased neuromuscular symptoms (1, 2), such as muscle weakness, muscle fatigability, generalized fatigue and joint and/or muscle pain (2–4). In most cases, these new symptoms are attributed to the development of post-polio syndrome (PPS) or described with broader terms that also incorporate symptoms more distantly related to the primary polio infection, such as “late onset polio sequelae” (4). New neuromuscular symptoms might further increase the already high risk of falling among persons with late effects of polio (5, 6), and limit physical mobility due to increasing problems with walking and climbing stairs (3, 7, 8). As a result, persons with late effects of polio experience challenges with their walking ability to avoid falling on a daily basis (9).

Recently, two 10-year follow-up studies reported that persons with late effects of polio significantly deteriorated in physical mobility (10) and functioning, mainly determined by increasing problems with indoor and outdoor walking as assessed with questionnaires (8). For daily indoor and outdoor walking, it is important to be able to adapt the gait pattern to environmental contexts and task goals to guarantee safe ambulation. We previously showed that this so-called walking adaptability (initially studied in persons after stroke (11)) is severely reduced for persons with late effects of polio in comparison with healthy individuals (12), and that this reduced walking adaptability is related to falls in this population (13). Since walking adaptability in persons with late effects of polio is mainly determined by the extent of leg-muscle weakness (13), which is known to decline gradually over time (10, 14–20), an age-related decline in walking adaptability can be expected, and might partly explain the increasing problems with daily-life indoor and outdoor walking in aging persons with late effects of polio.

No longitudinal studies on walking adaptability have been performed in persons with late effects of polio, but longitudinal studies that objectively assessed normal walking with timed walk tests reported significant, but modest, declines over follow-up periods ranging from 4 to 10 years (10, 15, 16, 21). Yet, timed walk tests are mostly performed under standardized conditions, while walking in daily life requires walking adaptability (11, 22), making it more challenging than normal walking. This could lead to a more pronounced decline in walking adaptability performance than normal walking performance. In healthy individuals, several cross-sectional studies showed that limitations in walking adaptability performance were more pronounced for elderly compared with younger adults (23–26), which may indicate a decline in walking adaptability over years but this has not been studies in persons with late effects of polio. Knowledge on the course of walking adaptability in persons with late effects of polio could be helpful to prevent falls and subsequent loss of independence in daily-life physical mobility.

According to Balasubramanian (11), walking adaptability can be conceptualized into 9 domains: obstacle negotiation, temporal demands, terrain demands, ambient demands, postural transitions, cognitive dual-tasking, motor dual-tasking, physical load, and manoeuvring during walking. However, not all of these domains can be easily and safely measured in clinical practice. Using the C-Mill interactive treadmill, we previously measured 2 of these domains, obstacle avoidance performance (mimicking obstacle negotiation) and variable target stepping (mimicking terrain demands such as walking on uneven surfaces), and found that both were severely reduced for persons with late effects of polio in comparison with healthy individuals (12). The aim of the current study is therefore to evaluate the course of variable target-stepping and reactive obstacle-avoidance performance over time as 2 important aspects of walking adaptability. Since walking adaptability course has not been studied before in persons with late effects of polio, a follow-up duration of 2 years was chosen to evaluate changes. It was hypothesized that walking adaptability would decline over the course of 2 years. In addition the study analysed the walking adaptability course in a subsample of participants diagnosed with PPS and exploratory subgroup analyses were performed to evaluate whether the 2-year course in walking adaptability performance would differ between subgroups stratified for baseline walking adaptability score, walking adaptability determinants in persons with late effects of polio, and self-reported fall frequency.

## METHODS

### Design and participants

This 2-year longitudinal follow-up study was performed at the outpatient clinic of the Department of Rehabilitation Medicine of Amsterdam UMC, location Academic Medical Center (AMC), The Netherlands. The study aimed to include 50 persons with late effects of polio (with or without PPS), who were aged between 18 and 80 years, reported at least 1 fall in the previous year and/or experienced fear of falling and were able to walk indoors without the use of walking aids (e.g. cane or stick). The diagnosis of PPS was made according to the March of Dimes criteria (2). Individuals diagnosed with other medical conditions directly associated with an increased fall risk (e.g. vestibular pathology, peripheral neuropathy and Parkinson’s disease) were excluded. All participants signed informed consent prior to study enrolment. The study protocol was approved by the Medical Ethics Committee of the AMC (reference number: 2016_159). Reporting of the study was in accordance with the Strengthening Reporting of Observational Studies in Epidemiology (STROBE) recommendations (27).

### Procedures

Four assessments were performed over the course of 2 years to assess walking adaptability: at baseline (T0), 2 weeks (T1), 1 year (T2) and 2 years (T3). The current study used T1, T2 and T3 measurements, and not T0 measurements because of known learning effects regarding walking adaptability (27). Therefore, we considered the T1 assessment as baseline. Information on the diagnosis PPS or not (obtained from the medical records), socio-demographic characteristics and manual leg-muscle strength were also assessed at baseline, and isometric leg-muscle strength, balance performance, fall frequency and fear of falling were collected at baseline, T2 and T3.

### Walking adaptability

**Fig. 1:**
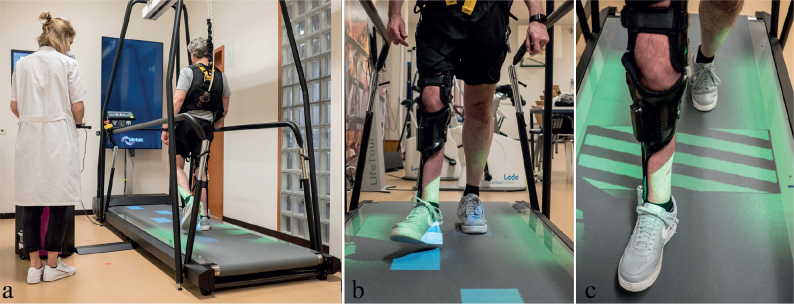
The C-Mill interactive treadmill. Walking-adaptability assessment on the C-Mill (*a*), with target-stepping tests (*b*) and obstacle-avoidance tests (*c*). Patient and staff permissions are given for this photo.

Walking adaptability was assessed on the C-Mill interactive treadmill (Motek, Amsterdam, the Netherlands, Fig. 1). At each measurement occasion, comfortable walking speed (CWS) was determined following Houdijk et al. (28). Subsequently, walking adaptability tests were performed at fixed CWS, starting with *target stepping* (in 3 conditions), followed by *obstacle avoidance* (in 2 conditions). Because of superior reproducibility, the most difficult target-stepping and obstacle-avoidance conditions were used for the current study (i.e. 30% variable target stepping and reactive obstacle avoidance, respectively) (27). In all conditions, handrail use was not allowed. In between tests, participants could rest while seated on a chair for at least 2 min until they felt recovered.

*Variable target-stepping performance.* Measured as the ability to align both feet with projected rectangular stepping targets (sized 3 cm longer than the participants right shoe) as good as accurately as possible. Trials started with 15 s of unconstrained walking to record the normal gait pattern, followed by 2 min target stepping in which step targets were projected with 30% random variation in imposed step length and step width relative to the normal gait pattern. For the calculation of target-stepping performance, all profiles of the centre of pressure (CoP) were inspected to check whether foot off and foot contact were localized at, respectively, the minima and maxima of the butterfly-shaped CoP-profile (29). We defined target stepping performance over goal-directed steps as the variable stepping error (VE, in mm), calculated as the standard deviation of all distances from the middle of the step target to the centre of the foot at midstance (12). A smaller VE indicates better target-stepping performance.

*Reactive obstacle-avoidance performance.* Defined as the ability to avoid 2D-obstacle projections on the treadmill belt during the swing phase at the estimated foot-placement location. Obstacles were successfully avoided when both feet were placed outside the obstacle boundaries. Obstacle-avoidance success rate (%) was scored through visual inspection and calculated over at least 10 correctly projected obstacles on the treadmill belt. A higher score represents better obstacle-avoidance performance.

### Leg-muscle strength

Leg-muscle strength was assessed manually according to the Medical Research Council (MRC) scale (range 0–5) (21) for 8 muscle groups per leg; hip abductors and adductors, hip flexors and extensors, knee flexors and extensors, and ankle plantar and dorsal flexors. Based on the MRC-sum score per leg (range 0–40), a most and least-affected leg were defined (i.e. lowest and highest MRC-sum score, respectively). In addition, isometric knee-extensor strength was assessed following a standardized procedure for both legs on a fixed dynamometer (Biodex system 4, Biodex Medical Systems, Shirley, NY, USA) while the back of the chair was positioned in 85° and the knee in 60° flexion. Maximal isometric strength was defined as the peak torque (Nm) of 3 maximal voluntary isometric contractions of 5 s, with 30 s rest in between contractions.

### Balance performance and balance confidence

Balance performance was assessed with the Berg Balance Scale (BBS) as the sum of 14 activity scores on a 5-point scale (range 0–4) (30). A lower BBS score indicates reduced balance performance, with a BBS score <46 as cut-off value for an increased risk of falling in elderly persons (31). The Timed-Up-and-Go test (TUG), shown to be reliable in persons with late effects of polio (32), was taken and scored as the time needed to rise from a chair, walk 3 m, turn around, walk back to the chair and sit down again (33). Balance confidence was assessed with the Activities Specific Balance Confidence (ABC) scale, a 16-item questionnaire in which confidence to maintain balance in different situations is scored (range 0–100%). A mean ABC score < 67% indicates an increased risk of falling in elderly persons (31).

### Fear of falling, falls and walking ability

Fear of falling was assessed with the short version of the Falls Efficacy Scale (short FES-I), a 7-item questionnaire in which fear of falling in different situations is scored, with reportedly sufficient measurement properties in persons with late effects of polio (34). A FES-I score (range 7–28) > 10 indicates increased fear of falling (35). A fall questionnaire was used to assess the number of falls in the previous year (none – one – two – three – four – five or more), self-reported level of functional ambulation (household – limited community – full community) and orthosis use (none – ankle-foot orthosis (AFO) – knee-ankle-foot orthosis (KAFO)).

### Statistical analysis

All analyses were performed in IBM SPSS Statistics for Windows, Version 26.0 (Armonk, NY: IBM Corp). Data were visually checked on normality, and descriptive statistics were used to summarize patients’ characteristics and clinical outcomes per measurement occasion. Baseline walking adaptability scores and clinical characteristics were compared between drop-outs and those who completed the study, using independent *t*-tests.

The 2-year course of walking adaptability performance was assessed with linear mixed model (LMM) analyses. Variable target-stepping performance and reactive obstacle-avoidance performance (independent variables) were analysed in separate models with a fixed effect of measurement occasion (dependent variable, represented by 2 dummy variables for time-points 2 and 3). LMM does not require imputation of missing values (36) and takes correlations between repeated observations into account by adding a random intercept at subject level.

As secondary, explorative analyses, this study analysed the walking-adaptability course in the subsample of participants who were diagnosed with PPS, and evaluated whether the walking-adaptability course differed between subgroups stratified for (*i*) baseline walking-adaptability score, (*ii*) known determinants of walking adaptability as recently shown in persons with late effects of polio (12), and (*iii*) self-reported fall frequency. Determinants of target-stepping performance in polio comprise leg-muscle strength, BBS score, ABC score and FES-I score, and, for obstacle avoidance, these comprise CWS, leg-muscle strength, BBS score, TUG score and ABC score (13). Per outcome, subgroups were created based on subjects’ baseline scores with low (i.e. ≤ median) vs high scores (i.e. > median), or on the number of falls reported in the previous year, with frequent fallers (i.e. ≥ 2 falls in the previous year) vs non-frequent fallers (i.e. ≤ 1 fall in the previous year). To determine whether the walking-adaptability course differed significantly (i.e. *p* < 0.05) between subgroups, the study evaluated the measurement occasion by subgroup interaction. For analyses regarding target-stepping performance, the study adjusted for CWS to account for individual changes in walking speed over time (37).

In an additional exploration, LMM-analyses were used to evaluate the 2-year course of factors previously shown to be associated with walking adaptability in persons with late effects of polio: CWS, leg-muscle strength, BBS score, TUG score, ABC score, FES-I score, and number of falls reported in the previous year.

## RESULTS

**Fig. 2:**
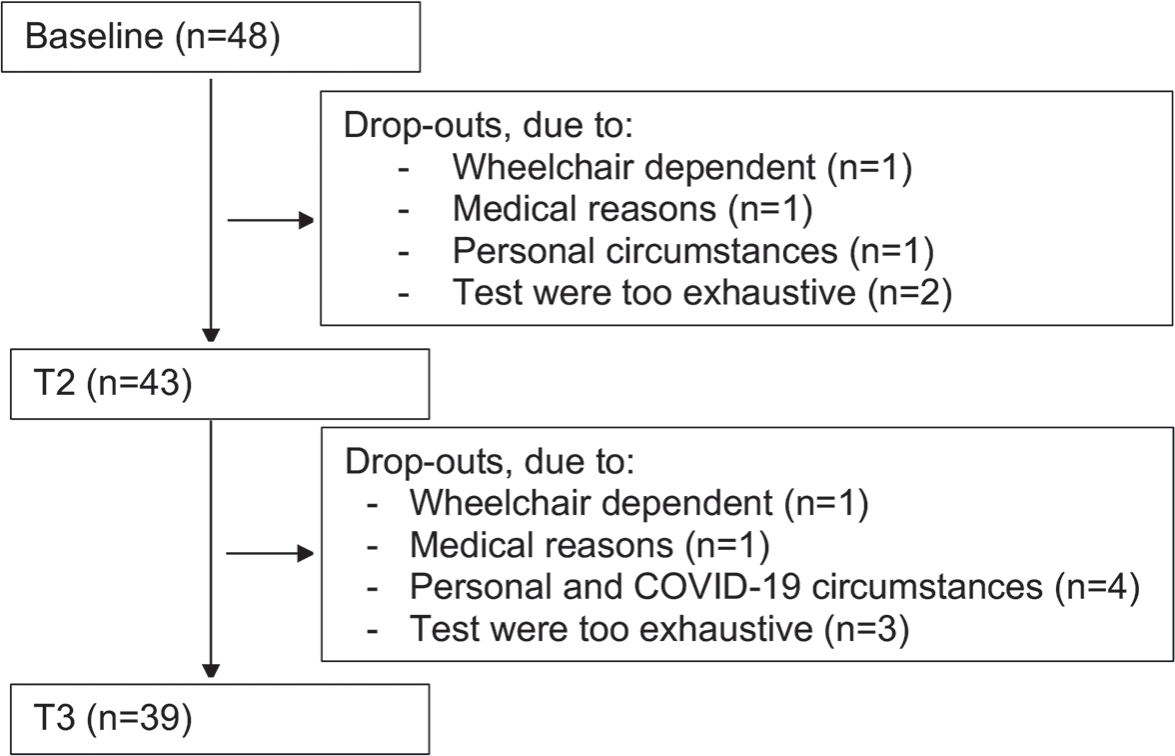
Flow-chart of participants.

From December 2016 to August 2018, 48 persons with late effects of polio were included in this study, of whom 41 were diagnosed with PPS. Baseline characteristics of these 48 participants are shown in Table I. Thirty-nine participants completed the 2-year follow-up assessment. A flow chart shows how participants progressed through the study, including reasons for dropout (Fig. 2). For walking-adaptability tests, there was some missing data due to invalid step detection during the target-stepping tests (*n* = 3 at each measurement occasion) and due to invalid obstacle-projections during the obstacle-avoidance tests (T1: *n* = 3, T2: *n* = 2 and T4: *n* = 1).

**Table I:** Participant’s characteristics, walking adaptability and clinical outcomes per measurement occasion

		Baseline (*n* = 48)	T2 (*n* = 43)	T3 (*n* = 39)
Sex (male/female), *n*		15/33		
Age (years), mean (SD)		63.1 (8.7)		
Mass (kg), mean (SD)		73.3 (12.7)		
BMI (kg/m^2^), mean (SD)		26.8 (4.3)		
Diagnosed with PPS (yes/no), *n*		41/7		
Walking-adaptability outcomes, mean (SD)				
CWS (m/s)		0.67 (0.28)	0.67 (0.26)	0.66 (0.23)
Variable target-stepping performance (VE, mm)		42.1 (9.5)	40.3 (8.9)	40. 5 (9.7)
Reactive obstacle-avoidance performance (%)		62 (27)	70 (27)	68 (24)
Clinical outcomes				
MRC-sum score (MA/LA-side) (median [IQR])		28 [4] / 38 [5]		
Isometric knee-extension strength, MA/LA-side, Nm, mean (SD)		35.6 (38.5)/94.0 (46.0)	40.1 (39.5)/87.2 (44.7)	38.1 (40.2)/94.5 (42.9)
BBS-score (mean (SD)		52.8 (3.3) Range: 44–56	52.3 (4.0) Range: 43–56	52.0 (4.7) Range: 36–56
TUG-score (mean (SD)		12.1 (3.0) Range: 7.7–21.0	12.4 (3.9) Range: 6.4–12.4	11.5 (2.8) Range: 6.7–17.8
ABC-score (mean (SD)		65.6 (20.1) Range: 19.4–100.0	64.4 (19.7) Range: 25.0–100.0	64.0 (20.3) Range: 26.0–99.4
Short FES-I score (mean (SD)		13.5 (5.6) Range: 7–23	13.5 (3.9) Range: 7–20	12.9 (3.8) Range: 7–23
Number of falls in the previous year (*n* (%))	Never	6 (13)	13 (30)	8 (21)
	Once	10 (21)	6 (14)	7 (18)
	Twice	6 (13)	8 (19)	7 (18)
	Three times	8 (17)	1 (2)	5 (13)
	Four times	4 (8)	2 (5)	2 (5)
	Five or more	14 (29)	13 (30)	10 (26)
Functional ambulation level (*n* (%))	Inside home	0	1 (2)	1 (3)
	In and around home	7 (15)	9 (21)	7 (18)
	< 1 km	30 (62)	22 (51)	23 (59)
	> 1 km	11 (23)	11 (26)	8 (20)
Orthosis use (*n* (%))	None	23 (48)	20 (47)	16 (41)
	AFO	10 (21)	10 (23)	11 (28)
	KAFO	15 (31)	13 (30)	12 (31)

T2: assessment at 1 year; T3: assessment at 2 years; BMI: body mass index; PPS: post-polio syndrome; CWS: comfortable walking speed; VE: variable stepping error; MRC: Medical Research Council; MA/LA: most-affected/least-affected; BBS: Berg Balance Scale; TUG: Timed-Up-and-Go; ABC: Activities Specific Balance Confidence; FES: Short version of the Falls Efficacy Scale; AFO: ankle-foot-orthosis; KAFO: knee-ankle-foot-orthosis; NA: not applicable.

### The 2-year course of walking adaptability

**Fig. 3:**
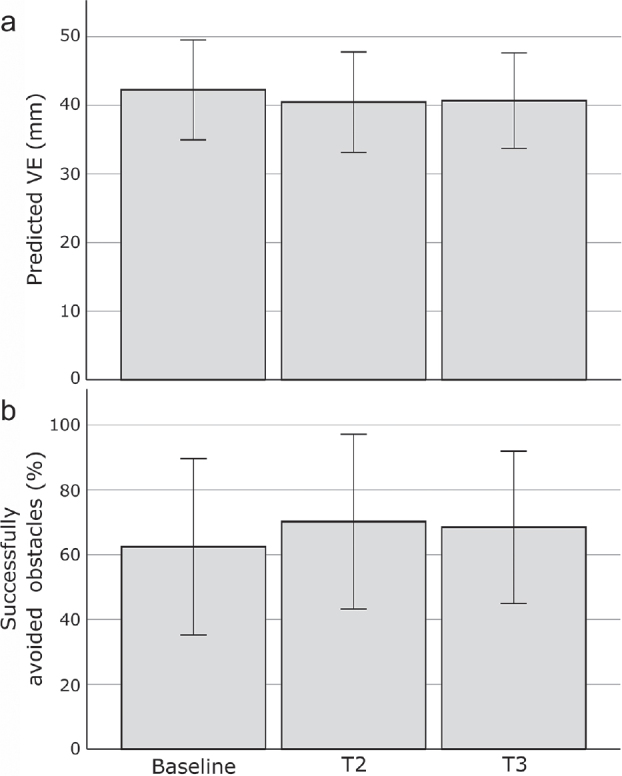
(a) The 2-year course of variable target-stepping performance. (b) The 2-year course of reactive obstacle-avoidance performance. For target-stepping performance (a), the VE (corrected for walking speed) is presented at baseline, 1 year (T2) and 2 years (T3). For reactive obstacle-avoidance performance (b), mean values for the percentage successfully avoided obstacles are presented for all measurement occasions. Error bars represent the standard deviation.

No changes were found in mean variable target-stepping and reactive obstacle-avoidance performance at 2 years from baseline (Fig. 3, Table II).

**Table II:** The 2-year course of variable target-stepping performance and reactive obstacle-avoidance performance

Outcome	Subgroup	One-year course (Baseline to T2)			Two-year course (Baseline to T3)		
		Estimate	95% CI	*p*-value	Estimate	95% CI	*p*-value
Variable target-stepping performance	None	–1.6	–4.0–0.7	0.165	–1.3	–3.8–1.1	0.285
Reactive obstacle avoidance performance	None	5.7	–2.1–13.5	0.147	4.2	–3.7–12.1	0.296
Variable target-stepping performance	PPS (*n = 43*)	–0.76	–3.2–1.7	0.531	–1.1	–3.6–1.5	0.413
Reactive obstacle avoidance performance	PPS (*n = 43*)	8.4	0.5–16.2	**0.037**	2.4	–5.7–10.4	0.557
Reactive obstacle avoidance performance Reactive obstacle avoidance performance	High BBS score *(n = 20)*	4.2	–7.2–15.6	0.465	13.6	1.9–25.4	**0.023**
	Low BBS score *(n = 28)*	7.0	–3.0–17.0	0.169	–3.6	–13.8–6.6	0.483

This table shows the main effect of measurement occasion on walking adaptability performance over the course of 2 years for the whole study population (upper part) and in a sample of participants diagnosed with post-polio syndrome (PPS) (middle part). The lower part of the table shows the differences in 2-year course in reactive obstacle-avoidance performance between subgroups stratified for low (≤ 54.0) vs high (> 54.0) Berg-Balance Scale (BBS) scores (based on median values). Target-stepping performance is given as the variable stepping error (VE, in mm), while reactive obstacle-avoidance performance is given as percentage successfully avoided obstacles. Significant values (*p*<0.05) are depicted in **bold**.

T2: assessment at 1 year; T3: assessment at 2 years; 95% CI: confidence interval.

### Explorative analyses

As indicated by the explorative analyses, walking-adaptability performance also did not change in 2 years in the subsample of participants diagnosed with PPS (Table II). Furthermore, it was found that the course in target-stepping performance (*p* = 0.271) and obstacle-avoidance performance (*p* = 0.085) were not significantly different for subgroups stratified for baseline walking-adaptability score. For walking-adaptability determinants, a significant interaction effect between measurement occasion and subgroups based on baseline BBS-score for reactive obstacle-avoidance performance indicated a significant improvement in obstacle-avoidance success rates for persons with late effects of polio with a high BBS-score (BBS > 54.0), but no significant change over time for those with a low BBS-score (BBS ≤ 54.0) (Table II). All other interaction effects were not significant. The course in target-stepping performance (*p* = 0.347) and obstacle-avoidance performance (*p* = 0.126) was not significantly different between frequent fallers and non-frequent fallers.

In our analysis on the course of factors previously shown to be associated with walking adaptability in persons with late effects of polio, a significant decline was found in isometric knee-extension strength of the least-affected leg from baseline to year 1 and a significant decline in BBS-score from baseline to year 2 (Table III). Other walking-adaptability determinants did not change significantly over time.

**Table III:** Two-year course of walking-adaptability determinants and self-reported fall frequency in persons with prior polio

	One-year course (Baseline to T2)			Two-year course (Baseline to T3)		
	Estimate	95% CI	*p*-value	Estimate	95% CI	*p*-value
Isometric knee-extension strength (MA-side)	–0.6	–3.4–2.5	0.757	–1.3	–4.3–1.8	0.416
Isometric knee-extension strength (LA-side)	–7.5	–14.4 to–0.6	**0.034**	–6.6	–13.8–0.7	0.076
BBS score	–0.8	–1.6–0.1	0.097	–1.1	–2.0 to –0.2	**0.022**
TUG score	0.6	–0.1–1.3	0.096	–1.2	–0.5–0.6	0.736
ABC score	–3.5	–7.8–0.9	0.118	–4.2	–8.7–0.3	0.068
Short FES-I score	0.03	–0.8–0.8	0.947	–0.1	–0.1–0.7	0.747
Walking speed	–0.02	–0.1–0.0	0.188	–0.01	–0.1–0.0	0.476
Self-reported fall frequency	–0.4	–1.0–0.1	0.106	–0.2	–0.1–0.3	0.389

Main effects of measurement occasion on clinical factors over the course of 2 years are shown. Significant values (*p*<0.05) are depicted in **bold**.

T2: assessment at 1 year; T3: assessment at 2 years; 95% CI: confidence interval; MRC: Medical Research Council; BBS: Berg-Balance Scale; TUG: Timed-Up-and-Go test; ABC: Activities Specific Balance Confidence; FES-I: short version of the Falls Efficacy Scale; LA: least affected; MA: most affected.

## DISCUSSION

This longitudinal study, of persons with late effects of polio with a known history of falls and/or fear of falling but with good balance performance, found no demonstrable change in walking adaptability, measured as variable target-stepping performance and reactive obstacle-avoidance performance over the course of 2 years. Explorative analyses showed that persons with late effects of polio with high balance performance scores improved their reactive obstacle-avoidance success rate over the course of 2 years, while reactive-obstacle-avoidance performance did not change for those with lower balance performance scores. Furthermore, the study found no difference in the walking-adaptability course between subgroups stratified for known walking-adaptability determinants in polio, which corresponds with the absence of any demonstrable changes in walking adaptability.

Walking adaptability did not change in our sample of persons with late effects of polio over a time period of 2 years. Since no other longitudinal studies have previously evaluated the walking-adaptability course in polio or in other clinical populations, these results cannot be compared. Yet, cross-sectional studies in community-dwelling adults reported reduced walking adaptability for older compared with younger adults (23–26, 38, 39), suggesting an age-related decline. In these studies, however, the age difference between the young adults (aged 20–37 years) and older adults (aged 65–88 years) was quite large. Possibly, 2 years may have been too short to capture changes in walking adaptability, as well as changes in clinical characteristics that are important for good walking adaptability in persons with late effects of polio (Table III) (40). Since muscle strength in persons with late effects of polio declines slowly, it might be that, in the current study, small changes in leg-muscle strength could not be measured because the measurement method used to assess muscle strength may not have been sensitive enough in this patient group (41, 42). This might also be the case for variable target-stepping performance, as we previously reported 95% limits of agreement that are considerably larger than the differences between measurement occasions found in the current study (27). Even though these differences are based on group mean values rather than individual values, target-stepping tests might be not sensitive enough to detect small changes over time.

Alternatively, since leg-muscle weakness is reportedly one of the best predictors for walking-adaptability performance in persons with late effects of polio (13), it might be that walking adaptability did not change because leg-muscle strength did not decline. Furthermore, a higher percentage of participants used an AFO to compensate for leg muscle weakness at 2 years follow-up than at baseline (Table I), which might have affected walking-adaptability performance. Yet, most likely, this resulted from participants without orthosis at baseline who dropped out during the study course, rather than from participants who started using an orthosis after baseline (i.e. only 2 participants used an AFO at 2-year follow-up while they did not use an orthosis at baseline). It therefore seems unlikely that changes in orthosis use influenced our outcomes. Thus, since muscle strength is an important predictor of walking adaptability, but did not decline over the course of 2 years, and, considering that walking adaptability is associated with falls (13), which were reported in all of our participants, the role of other fall-risk factors, such as cognition, reactive balance control, and reaction time (43, 44) should be evaluated in future research to establish a more complete view of walking adaptability in persons with late effects of polio. Furthermore, on the C-Mill interactive treadmill, walking adaptability was elicited by 2D projections, while obstacles in daily life are mostly 3D. Future research should incorporate such daily-life environmental circumstances (i.e. 3D obstacles and ambient demands) when assessing walking adaptability in persons with late effects of polio.

While we previously identified several clinical factors that might account for the reduced target-stepping performance and reactive obstacle-avoidance performance in persons with late effects of polio (13), this study found hardly any significant differences in walking-adaptability course between subgroups stratified for these factors. We created subgroups based on median scores rather than on published cut-off values to guarantee an even distribution of participants over subgroups. As a consequence, between-group contrasts might have been low, which reduced the likelihood of significant between-group differences. Despite these small contrasts, an explorative analysis revealed that obstacle-avoidance performance improved significantly for persons with high balance performance, while it did not change for those with lower balance performance. This finding might be related to the relevance of good balance skills required when avoiding suddenly appearing obstacles (13), and, in this context, balance training might be an important means to maintain or improve balance skills in persons with late effects of polio as to maintain their walking adaptability performance over time. However, note that balance performance as assessed with the BBS in the current study population was rather high at baseline (i.e. only 2 participants scored below the cut-off value of 46) and that it was still high after 2 years (i.e. only 5 participants scored below the cut-off value of 46; Table II), probably explained by previously reported ceiling effects for this test (27). Yet, because of the rather slow progression of the late onset polio sequelae, the sensitivity of a measurement tool is important to detect small changes over time in this population. This might challenge the clinical implications of the current results regarding the interaction with balance performance, and future longitudinal studies with a longer follow-up should further elaborate on the effect of balance impairments on the time course of reactive obstacle-avoidance performance in persons with late effects of polio.

When interpreting the current study results, it is important to consider some limitations in the study design and set-up. Walking adaptability in this study was assessed on the C-Mill interactive treadmill. C-Mill system updates during the 2-year study period might have improved estimated foot-placement localization and timing, leading to a better tuning between object projections and foot placement. In general, software updates are inevitable, but in our study they could have affected the feasibility of the walking-adaptability tests and might therefore have overestimated walking-adaptability performance at the 2-year follow-up measurement. Furthermore, some participants were lost during follow-up due to medical reasons, the SARS-CoV-2 (COVID-19) pandemic or because tests were too exhaustive, which also could have resulted in an overestimation of walking-adaptability outcomes and clinical characteristics at 2 years. Yet, since baseline walking-adaptability scores and clinical characteristics between drop-outs and those who completed the study did not differ significantly, except for a significantly higher FES-I score for drop-outs, we expect this effect to be small. This study was the first that longitudinally evaluated the course of walking adaptability in persons with late effects of polio over time, and we therefore performed some additional explorative analyses to evaluate the effect of clinical factors on the course of walking adaptability. Since these analyses were performed on the same data, we should interpret the results with caution to prevent type I errors. For future studies, we would recommend to extend the follow-up period of the study to at least 4 years (40), include persons with late effects of polio without a fall history and/or fear of falling in order to increase between-group contrasts, use a prospective instead of retrospective fall registration to prevent recall bias, and include a control group consisting of people without polio or other gait-and-balance-disorders in order to put the current results into perspective.

In conclusion, this 2-year longitudinal study, on walking-adaptability course in persons with late effects of polio with a history of falls and/or fear of falling but with good balance performance, did not find demonstrable changes in variable target-stepping and reactive obstacle-avoidance performance. This may be due to insufficient sensitivity of the outcome measures to detect small changes over time, or the absence of a decline in clinical characteristics known to be associated with walking adaptability in this population, such as leg-muscle strength. Since leg-muscle strength is one of the best predictors for walking adaptability in polio, and considering the high number of studies that reported a decline in leg-muscle strength over time among persons with late effects of polio, but not within 2 years, future longitudinal studies on walking adaptability in this population should extend their follow-up period to at least 4 years in order to re-evaluate the current findings.
